# Nucleophosmin: from structure and function to disease development

**DOI:** 10.1186/s12867-016-0073-9

**Published:** 2016-08-24

**Authors:** Joseph K. Box, Nicolas Paquet, Mark N. Adams, Didier Boucher, Emma Bolderson, Kenneth J. O’Byrne, Derek J. Richard

**Affiliations:** School of Biomedical Research, Institute of Health and Biomedical Innovation at the Translational Research Institute, Queensland University of Technology, Brisbane, QLD Australia

**Keywords:** Nucleophosmin 1, DNA repair, Cancer, Apoptosis

## Abstract

Nucleophosmin (NPM1) is a critical cellular protein that has been implicated in a number of pathways including mRNA transport, chromatin remodeling, apoptosis and genome stability. NPM1 function is a critical requirement for normal cellular biology as is underlined in cancer where NPM1 is commonly overexpressed, mutated, rearranged and sporadically deleted. Consistent with a multifunctional role within the cell, NPM1 can function not only as a proto-oncogene but also as a tumor suppressor. The aim of this review is to look at the less well-described role of NPM1 in the DNA repair pathways as well as the role of NPM1 in the regulation of apoptosis and its mutation in cancers.

## Background

**Fig. 1:**
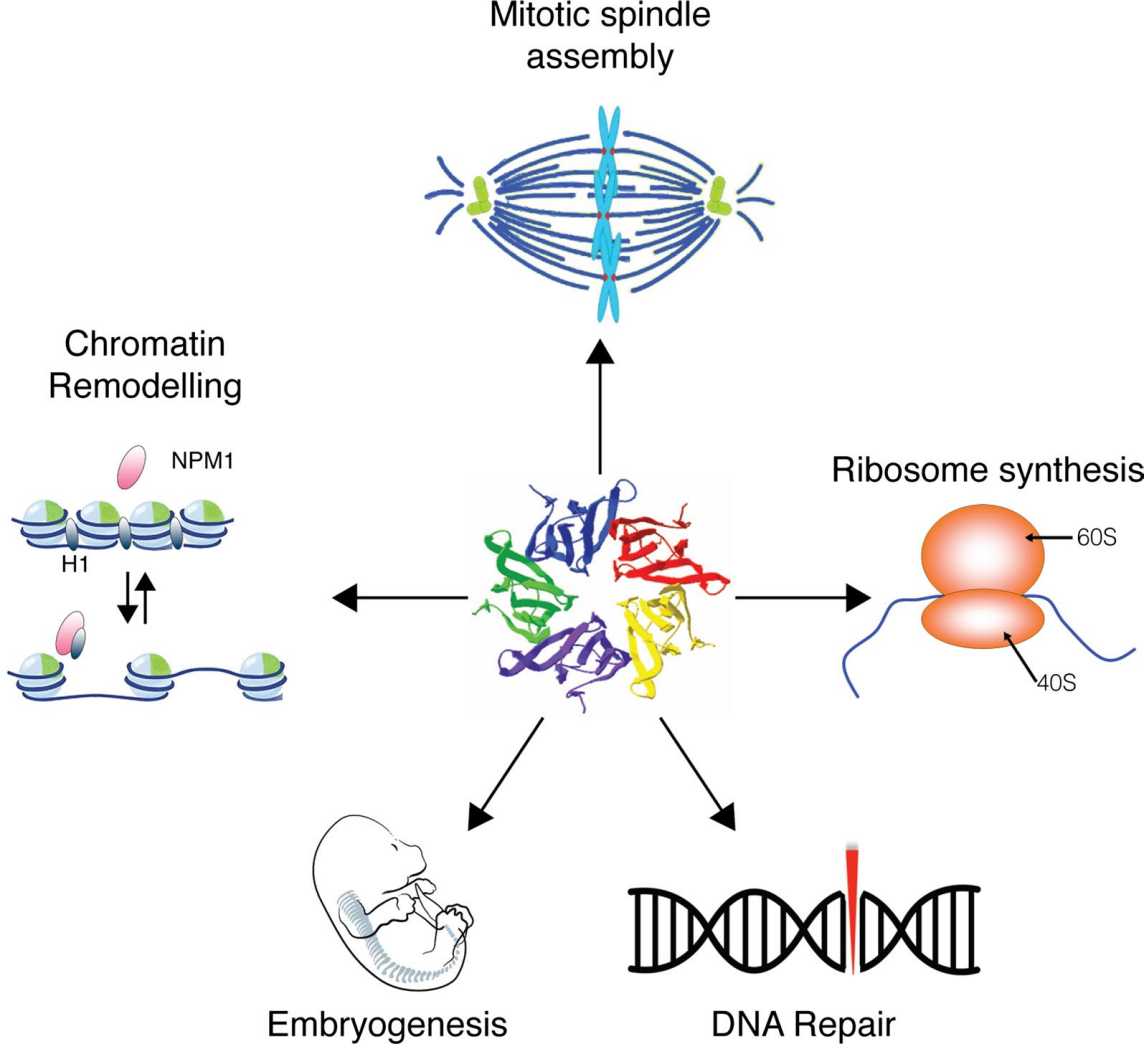
Overview of NPM1 functions within cells. NPM1 participate to many biological processes such as DNA repair, embryogenesis, likely by interacting with the chromatin, by binding to histones and other chromatin remodeling proteins. Importantly, NPM1 also promotes ribosome biogenesis

Nucleophosmin (NPM1), also known as B23, No38 or Numatrin, is an abundant nucleolar protein found in the nuclei of proliferating cells. NPM1 has been documented as participating in ribosome biogenesis, mRNA processing, chromatin remodeling, and embryogenesis (Fig. 1). While much is known about the function of NPM1 in metabolic pathways, it is becoming clear that NPM1 also has a critical function in maintaining genomic stability by functioning in various DNA repair pathways and regulating apoptosis. In this review, we shall present an updated overview of these roles, in particular the emerging data supporting a role for NPM1 in DNA repair. Lastly, we shall look at how NPM1 dysfunction contributes to cancer pathologies.

### Gene organization and evolutionary history

**Fig. 2:**
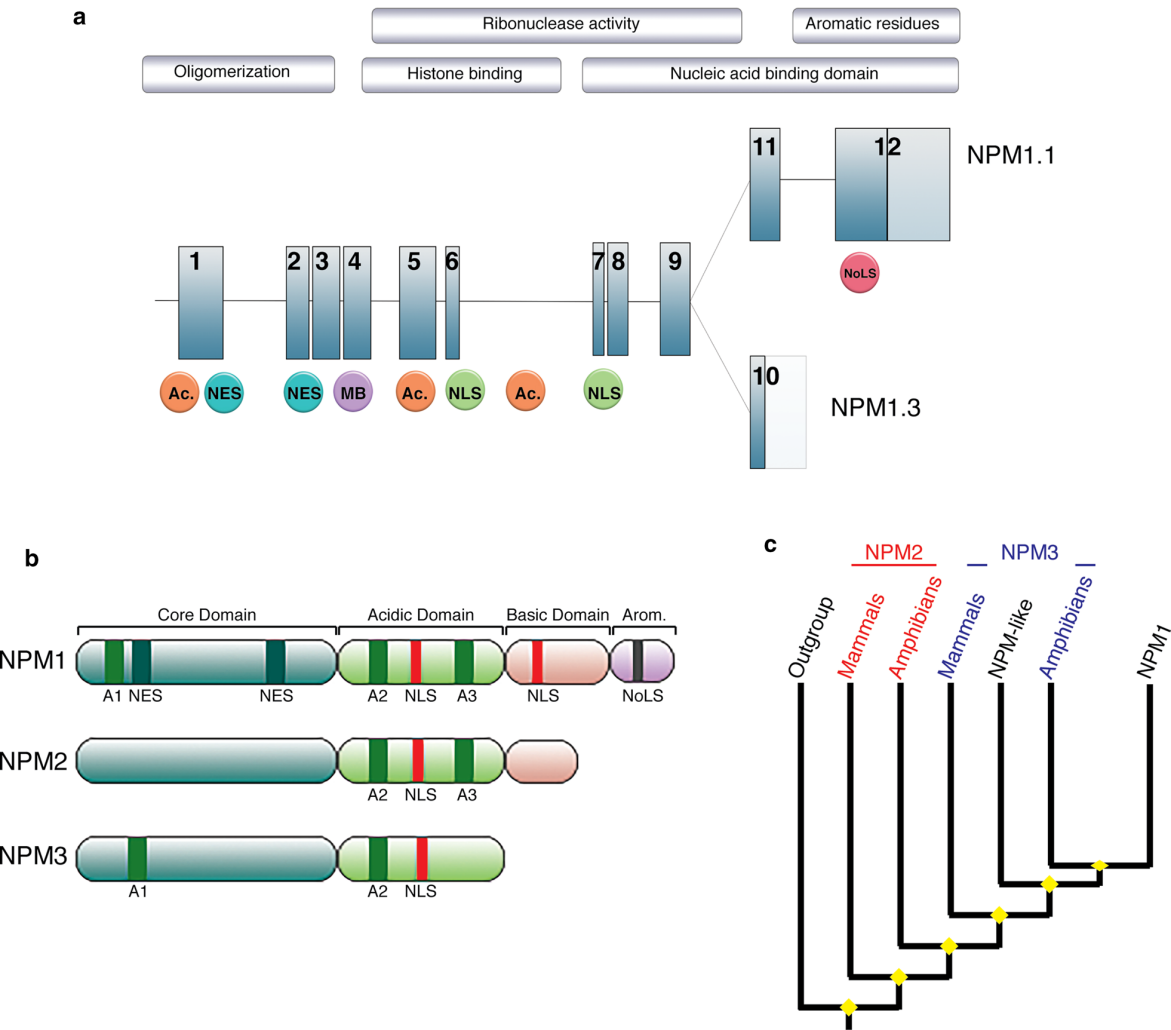
**a** Superposition of NPM1 genomic structure and protein features. NPM1.1 and NPM1.3 are two splices variants resulting from the use of alternative codons. The two isoforms have different expression levels and localization. *Ac* Acidic domain, *NES* nuclear export signal, *MB* putative metal binding domain, *NLS* nucleus localization signal, *NoLS* Nucleolar localization signal. **b** Schematic structure of NPM proteins from human (not to scale). All proteins share a core, hydrophobic domain (*blue*) responsible for oligomerization and chaperone activity, followed by an acidic domain required for ribonuclease activity. A basic domain implicated in nucleic acid binding is common to NPM1 and NPM2, but absent in NPM3. Finally, only NPM1 exhibits a C-terminal aromatic stretch require for its nucleolar localization. In addition, NPM members harbor nuclear-localization signals (NLS), nucleolar-localization signal (NoLS), nuclear export signal (NES) and acidic clusters (**a**). **c** Simplified representation of the phylogenetic relationship within the NPM family. Inferred from [4]. The dendrogram reveals a clustering of sequences by type, rather than by species, and identifies NPM2 and NPM3 as polyphyletic groups

Human *NPM1* is located on chromosome 5q35 and is composed of 12 exons, encoding for at least two isoforms (Fig. 2a) [1]. NPM1.1 (or B23.1), corresponding to the full-length transcript, results in a 294 amino acids protein (35–40 kDa) abundantly expressed in all tissues. Alternatively, NPM1.3 (also known as B23.2) results from the use of a distinct 3′ exon, and encodes for a protein expressed at low levels in cells, lacking the last 35 amino acids of the NPM1 C-terminus [2]. A third isoform NPM1.2 has been suggested, but so far, no biological data support this finding [3].

NPM1 belongs to a histone chaperones family, the Nucleophosmin/nucleoplasmin (NPM) family, a group that comprises multiple major functional members (NPM1, NPM2, NPM3 and the invertebrate NPM-like), and can be found amongst all Metazoan [4]. While this family is well characterized functionally, little is known about the evolution of these genes and proteins. At a glance, all members of the NPM family exhibit conserved structural motifs; a N-terminal core domain, an acidic domain and a nuclear localization signal, associated with a less conserved, disorganized C-terminus region (Fig. 2b) [5]. Subsequently, crystallographic studies revealed a similar tertiary organization for NPM1 and NPM2 with monomers organized into pentameric donut-shaped complexes [6, 7].

As simplified in Fig. 2c, phylogenetic analysis revealed the late emergence of the NPM1 monophyletic clade while, in contrast, NPM2 and NPM3 lineages appear of polyphyletic origin, with mammalians and amphibians sequences clearly differentiated [4]. Consistent with their shared expression profile, localization and direct physical interaction, NPM1 and NPM3 are the most closely related members of the family, suggesting functional constraints between the two proteins [4]. Huang et al. [8] further suggest that NPM3 may have evolved following the loss of a nucleic acid binding domain of NPM1, and functions as an element regulating NPM1 RNA binding.

Interestingly, codon usage within the NPM gene family indicates a strong purifying selection, materialized by a high rate of silent mutations which significantly deviates from neutrality. The highly conserved organization of NPM proteins as pentamers further supports the hypothesis of a strong negative selection operating at the structural level. Interestingly, potential sites of post-translational modifications are also selectively constrained, being conserved not only at the protein level but also showing a preferred codon usage [4]. Remarkably, these characteristics are shared with evolutionary features observed in histones, suggesting intertwined evolutionary history between the two families [9].

Despite being the most recent divergent NPM lineage, the functions of NPM1 are diverse and include roles in ribosome biogenesis [10, 11], mRNA processing [12], chromatin remodeling [13], embryogenesis [14], regulation of apoptosis and maintenance of genome stability.

### Characteristic structural features of nucleophosmin

NPM1 structural architecture is well characterized by three distinct regions, onto which nucleolar and nuclear localization motifs, nucleic acids binding domains, oligomerization domains, histones binding regions, as well as a putative metal binding domain, have been mapped and described in detail [15–17] (Fig. 2a, b).

**Fig. 3:**
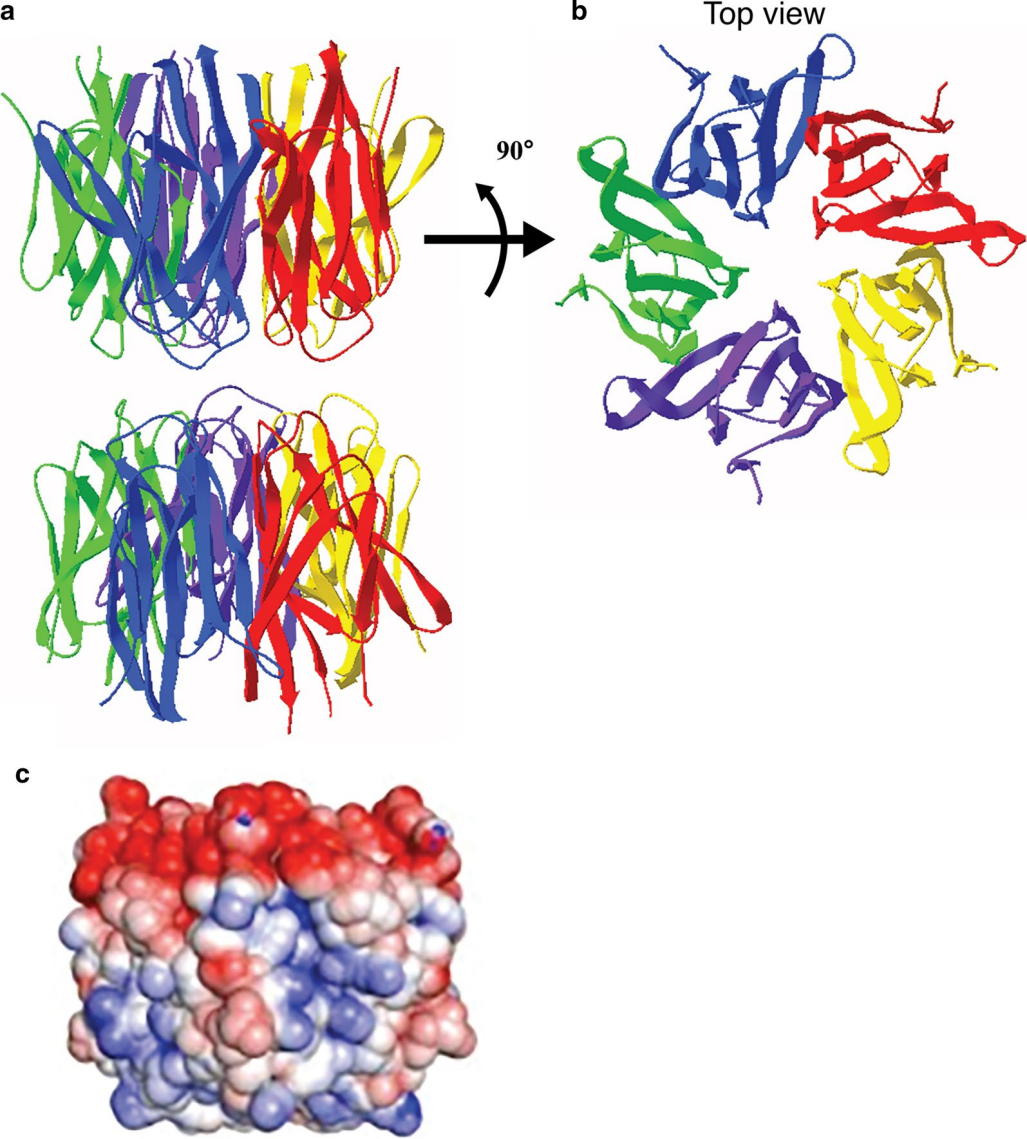
**a** The crystal structure of the N-terminal oligomerization domain of NPM1 organized in a decamer, with two NPM1 pentameric rings stacked in a head-to-head fashion. Each NPM1 monomer is depicted in a *different color*. Structure derived from PDB data (PDB ID: 2P1B; [18]) visualized using Swiss PDB viewer. **b** Top view of a pentameric structure shown in **a**, showing the organization of monomers into a *donut shaped ring*. **c** Electrostatic potential map of **a** and **b**, showing an asymmetric distribution of charges, with negative charges (*red*) on the top surface of the pentamer and neutral (*white*) to slightly positive (*blue*) charges

The N-terminal region is highly conserved in all members of the Nucleoplasmin/Nucleophosmin family and constitutes the core domain, which mediates NPM1 oligomerization and interactions with other proteins. The three dimensional structure of the human NPM1-core has been determined by X-ray crystallography and showed an organization into eight β-barrels forming a jelly roll barrel. Further, NPM1 monomers associate as donut-shaped homo-pentamers (Fig. 3a, b). The distribution of charges in this region is extremely asymmetric with negatively charged residues clustered on one side of the oligomer. Two pentamers of NPM1 interact in a head-to-head manner to form a decamer, and are arranged so that a monomer of the pentameric ring only contacts a single monomer of the other pentamer, allowing structural plasticity at the pentamer–pentamer interface [18]. This multimeric state is modulated by numerous post-translational modifications, especially phosphorylation events that regulate the monomer-pentamer equilibrium by promoting the disassembly of the pentamer into unstable, unfolded monomers. This structural polymorphism participates in the regulation of NPM1 localization and function [6]. As such, oligomerization of NPM1 has been linked to its nucleolar localization and role in cellular proliferation, while the monomeric form of NPM1 is associated with its role in the DNA damage response and induction of apoptosis [19].

The NPM1 central region appears unstructured and is marked by the presence of highly acidic regions composed of strings of aspartic and glutamic acids (A1, A2 and A3). They provide long tracks of negatively charged residues, known to be involved in the binding to Histones H1, H3, H4, H2A and H2B, potentially by mimicking the charges of DNA and RNA [20, 21]. It also contains a nuclear localization signal.

The C-terminus of NPM1 is characterized by the presence of a basic, positively charged cluster of amino acids, immediately followed by a stretch of aromatic residues, providing an adequate platform allowing the binding to nucleic acids and ATP [16, 22]. These aromatic residues constitute an atypical nucleolar localization signal (NoLS), and their mutation are responsible for the unfolding and the aberrant NPM1 localization typical in acute myeloid leukemia (AML) cases.

### Function of NPM1 in apoptosis

Although the best described function of NPM1 is in ribosome biogenesis, NPM1 also displays a critical role in regulating apoptosis. NPM1 expression levels have been implicated in controlling the cellular apoptotic response. In a variety of cell based models, several studies have demonstrated that down-regulation of NPM1 sensitizes cells to apoptosis, while increased levels of the protein protects against apoptosis [23–25]. In a disease setting, the balance between NPM1 expression and cell fate is demonstrated in hypoxia-driven cancers. For example, suppression of hypoxia-induced NPM1 expression promotes apoptosis whereas overexpression protects from hypoxia-mediated cell death [26]. From a cancer perspective, elevated levels of NPM1 might promote malignant transformation by enabling cell survival.

**Fig. 4:**
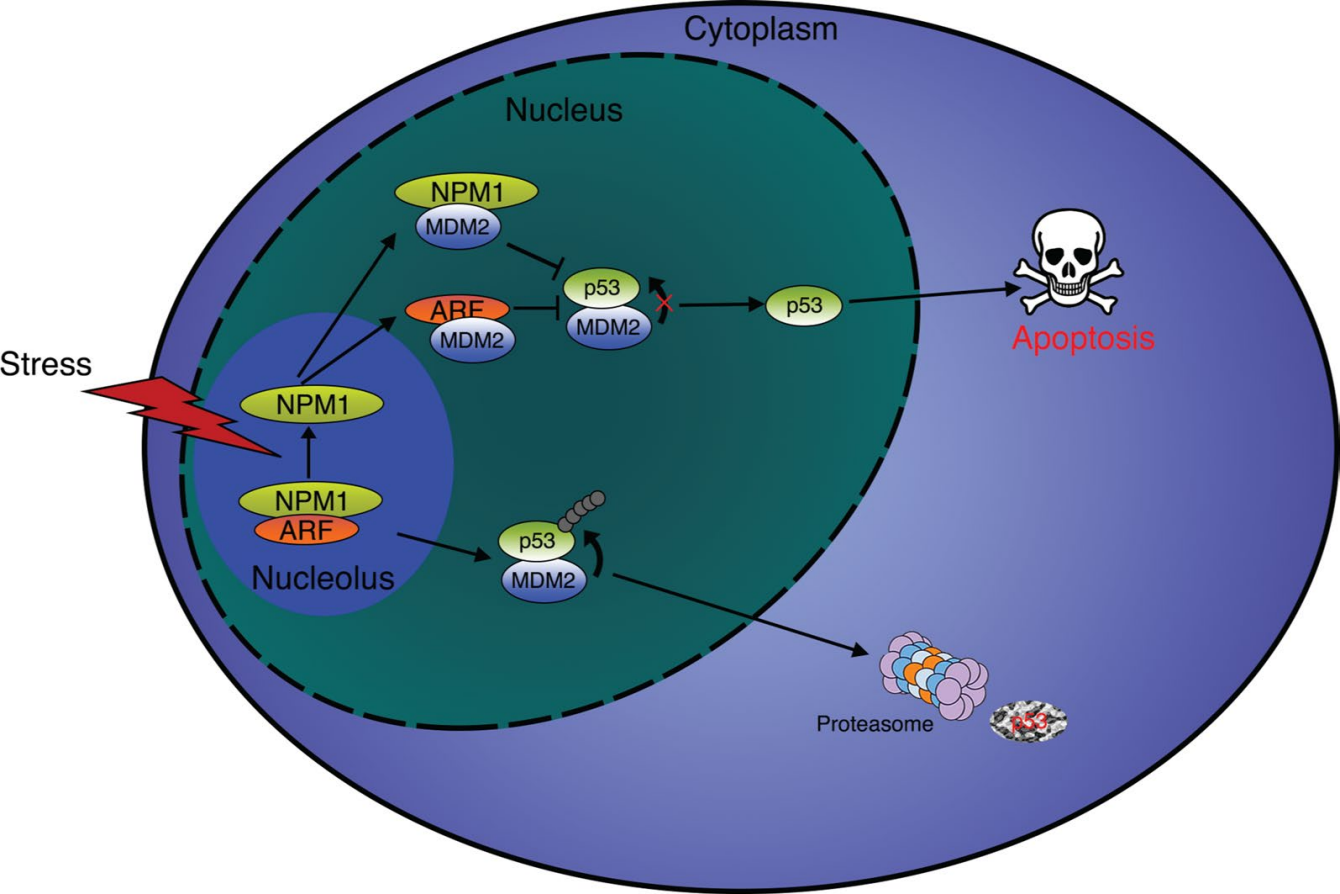
Regulation of apoptosis by NPM1. In unstressed cells, p14^ARF^ and NPM1 form a dimer in the nucleoli, allowing MDM2 to target p53 for proteasomal degradation. Following a stress, such as DNA damages, p14^ARF^ and NPM1 dissociate and relocate to the nucleus were they sequester MDM2, leading to the stabilization and activation of p53. p53 then induces the transcription of various genes involved in cell-cycle arrest, DNA repair and apoptosis

Several proteins have been identified that impact cell survival by interacting with and regulating NPM1 protein levels. In the nucleolus, the tumor suppressor p14^ARF^ interacts with NPM1 to promote degradation of the protein and induce cell death (Fig. 4) [27]. However, the interplay between NPM1 and p14^ARF^ is more complex, with NPM1 also acting as major cellular reservoir of p14^ARF^ (Fig. 4). After various stimuli NPM1 releases p14^ARF^ allowing binding to MDM2 and preventing the proteasomal degradation of p53 (Fig. 4) [28]. Consistently, depletion of NPM1 with siRNA results in increased apoptosis due to a greater amount of free p14^ARF^ [28]. On the contrary, up-regulation of NPM1 appears to antagonize p14^ARF^ and increases its nucleolar retention [29]. These observations provide evidence that NPM1 regulates cell fate in a p53-dependent manner by directing p14^ARF^ to nucleoli and preventing inhibition of MDM2 (Fig. 4) [27, 30, 31]. Other studies have also linked NPM1 with the tumor suppressor activity of p53. For example, NPM1 is also able to interact directly with MDM2, independently of p14^ARF^, and act as a p53:MDM2 inhibitor to protect p53 from degradation (Fig. 4) [32, 33]. Interestingly, NPM1 is also reported to directly associate with p53 [34]. However, this interaction remains contentious as an inability for NPM1 to interact with p53 has also been reported [27]. It may be that an interaction between NPM1 and p53 might only occur in certain cellular contexts. Nonetheless, this observation requires clarification.

In addition to p14^ARF^, MDM2 and possibly p53, NPM1 has been reported to bind PKB/Akt in order to modulate cell survival. In the nucleus, Akt binds NPM1 in response to growth factor stimulation to protect NPM1 against caspase-3-mediated proteolytic degradation and promote cell survival [35]. Similarly, NPM1 stability is enhanced by interacting with erythroid differentiation-associated gene (EDAG), promoting acute myeloid leukemia (AML) cell survival [36]. In another instance, the interaction of GAGE with NPM1 also enables NPM1 stability to promote resistance to interferon-γ-induced apoptosis [37].

NPM1 has also been portrayed as regulating cell fate by modulating both the intrinsic and extrinsic apoptosis pathways. During the intrinsic apoptotic response, p53 is required in the mitochondrial to enable cytochrome C release. Overexpression of NPM1 prevents the translocation of p53 from the nucleus to the mitochondria [38], suggesting that NPM1 may protect cells from apoptosis by reducing the mitochondrial level of p53. In acute promyelocytic leukemia cells expressing the NPM1-retinoic acid receptor α (NPM1-RAR) fusion protein, NPM1-RAR blocked TNF-induced extrinsic apoptosis by inhibiting signaling to activate caspase-3 and -8 [39]. Similarly, mutant forms of NPM1 are indicated to impede apoptosis by directly inhibiting the proteolytic function of caspase-6 and -8 in the cytoplasm [40]. Interestingly, in anaplastic large-cell lymphoma cells, the cytoplasmic fraction of the NPM1-ALK fusion protein is solely responsible for inducing apoptosis by engaging the DNA-damage response [41]. These studies suggest, at least for the mutant or NPM1 fusion proteins, that the cytoplasmic fraction of NPM1 may be required to regulate the apoptotic pathways.

### Role of NPM1 in the DNA repair response

Loss of NPM1 function has been shown to be associated with increased genome instability [42]. Several studies have demonstrated the critical role of NPM1 in the maintenance of genome stability through its interaction with unduplicated centrosomes [42]. The phosphorylation of NPM1 by CDK2/Cyclin E promotes the release of NPM1 from the centrosome during duplication; this represents an essential step for duplication to occur. However, during mitosis NPM1 re-associates with the centrosomes at the spindle bodies and appears to be controlling centrosome duplication [43]. Indeed, depletion of NPM1 has been shown to promote genome instability (unaligned chromosomes, micronuclei) [44, 45]. In mice, the chromosomal instability associated with NPM1 depletion partially explains the embryonic lethality [14].

**Fig. 5:**
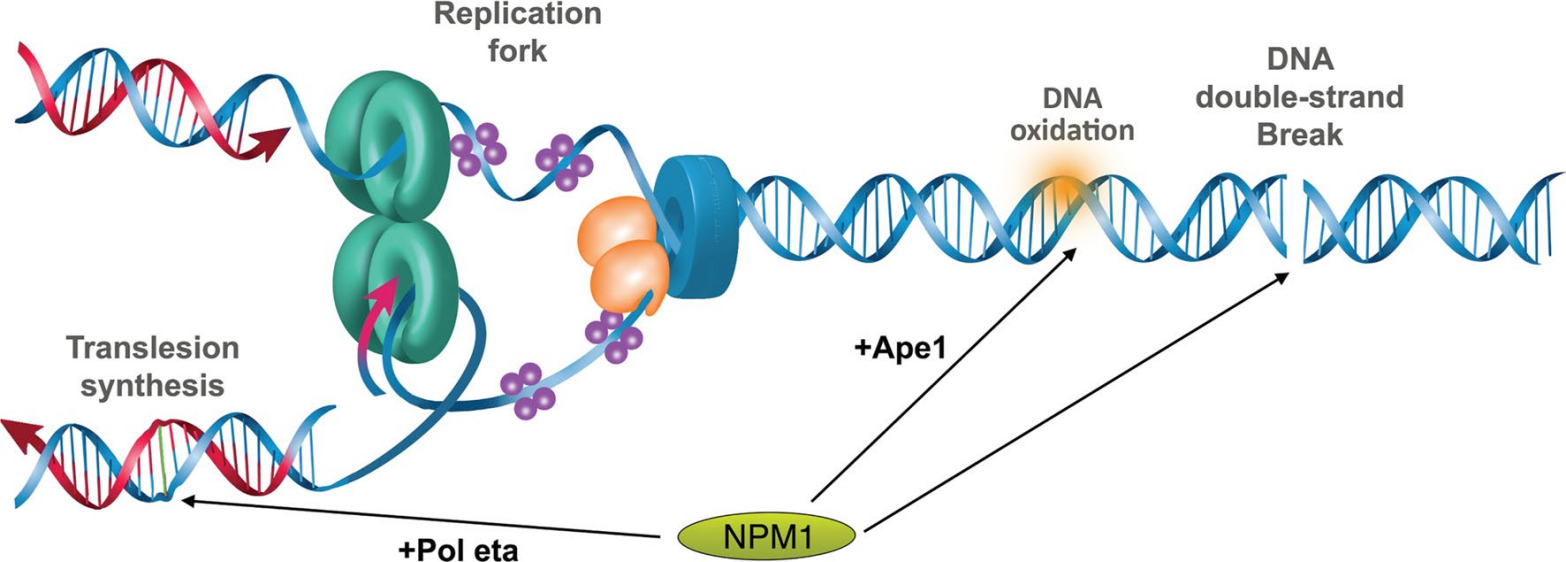
Overview of NPM1 functions in various DNA damage response pathways. NPM1 has been shown to modulate the BER pathway as well as the translesion synthesis by modulating the levels of apurinic/apyrimidinic endonuclease 1 (APE1) and polymerase eta. In the absence of NPM1 or with the expression of non-phosphorylatable NPM1, double-strand break repair by homologous recombination fails to be completed, however the exact mechanism of NPM1 function in these pathway remain to be fully elucidated

However, it has only recently become clear that NPM1 is likely to have a direct role in the repair of DNA lesions. Multiple DNA repair pathways promote the repair of different DNA lesions and NPM1 has been implicated in several of these repair pathways (Fig. 5).

DNA double-strand breaks (DSB) are the most destructive and genotoxic lesions encountered by cells and as such complex cascades have evolved to sense, signal and repair these breaks. DNA DSBs can then be repaired by two pathways, homologous recombination (HR) and non-homologous end-joining (NHEJ) (reviewed in [46]). HR utilizes a sister chromatid as a template for repair and therefore can only be performed in the late S-phase and G2 phases of the cell cycle. In contrast, NHEJ can occur in any phase of the cell cycle and involves a less complex method of ligating the two DNA ends together. As overhanging DNA may be resected during this method, NHEJ is generally known as the more error-prone mechanism of DNA DSB repair. In the HR process, DSBs are detected and signaling pathways are initiated by the ATM/ATR kinases, promoting the recruitment of repair proteins, including nucleases that resect DNA with a 5′–3′ polarity. This resection generates stretches of single stranded DNA, which invade into a sister chromatid, allowing it to act as a template for polymerase-mediated extension of the invading strand. Following this extension and re-ligation of DNA strands this reaction then yields two intact and identical DNA molecules [47].

Thus far, NPM1 has only been implicated in homologous recombination (HR). Following DNA double-strand break induction, NPM1 is recruited from the nucleolus into the nucleoplasm and binds to the chromatin [48]. NPM1 is phosphorylated at Threonine 199 by the cyclin-dependent kinases CDK1 and CDK2 during the cell cycle [49]. A subsequent study showed that following induction of DNA double-strand breaks by ionizing irradiation, NPM1 phosphorylated on threonine 199 localizes to sites of DNA double-strand breaks, colocalizing with other DNA repair proteins such as γH2AX and BRCA1 [50]. Phosphorylated NPM1 is recruited to sites of DNA marked by K63-linked ubiquitin conjugates in a process dependent upon the ubiquitin ligases RNF8 and RNF168. Depletion of NPM1 or expression of non-phosphorylatable NPM1 (T199A) leads to persistence of Rad51 foci suggesting that repair of DNA DSBs is not completed. This is further supported by the detection of increased DNA lesions in cells expressing NPM1 T199A. In contrast, cellular survival following ionizing radiation was not significantly affected [19]. Another study also analyzed γH2AX and Rad51 foci kinetics and showed that DNA damage also persisted in NPM1-null mouse embryonic fibroblast (MEF) cells [51]. Like the studies in human cells, NPM1-null MEFs also exhibited increased DNA lesions and a modest decrease in cellular survival following ionizing radiation. An anti-cancer agent, YTR107, was also shown to act by binding to NPM1 and altering its function. YTR107 inhibits DNA repair and radiosensitizes cells in an NPM1-dependent process [51]. In light of the above it is clear that NPM1 has a role in DNA DSB repair, however much is still to be elucidated about the exact mechanism of its function in these pathways (Fig. 5).

Following exposure of cells to agents such as UV, DNA lesions may cause replication forks to stall as the replicative polymerases are unable to bypass the UV-induced bulky lesions. In order for these stalled replication forks to be restarted, they can be repaired by a form of HR or be bypassed in a mechanism known as translesion synthesis (TLS, reviewed in [52], Fig. 5). A recent study identified NPM1 as a key player of the TLS pathway [53]. This process enables switching of the DNA polymerase to a low fidelity DNA polymerase that can replicate the DNA across the lesion. NPM1 regulates TLS by binding to and protecting DNA Polymerase Eta (POLH, polη) from proteosomal degradation promoting its role in TLS. The mutated NPM1 (NPM1c+, found in 30 % of AML cases) was found to result in increased degradation of polη, perhaps explaining the improved prognosis in AML patients with NPM1 mutations [53].

In addition to its role in DNA double-strand break repair and translesion synthesis, NPM1 has also been shown to respond to DNA lesions induced by UV light. The alterations to nucleotides caused by UV irradiation are repaired by the nucleotide excision repair pathway, a process that is dependent upon the PCNA homo-trimer (reviewed in [54]). The levels of NPM1 protein were shown to increase following cellular exposure to UV [55]. Exogenous overexpression of NPM1 was also found to increase cellular survival and DNA repair capacity following UV irradiation. Supporting a role for NPM1 in nucleotide excision repair (NER), it was found to transcriptionally regulate the crucial NER protein PCNA [55]. Following UV irradiation, dephosphorylation of NPM1 on Threonine 199, 234 and 237 residues occurs in a PP1β-dependent manner. Dephosphorylation of these sites on NPM1 enhances the interaction between NPM1 and the retinoblastoma tumor suppressor protein (pRB), which then allows the release of E2F1 from pRB. E2F1 subsequently functions to transcriptionally activate several downstream DNA repair genes, including XPC, DDB2 and RPA14, facilitating DNA repair [56].

Oxidative damage to DNA is caused predominantly by normal cellular metabolism and is repaired by the base excision repair pathway (BER reviewed in [57]). NPM1 has been shown to modulate the BER pathway through control of the apurinic/apyrimidinic endonuclease 1 (APE1) protein levels and modulation of the AP-site incision activity of APE1, which is required for base excision repair (Fig. 5) [58–60]. Several nucleolar proteins involved in BER were also mislocalised in NPM1-deficient cells, including APE1, Fen1 and LigI [61]. NPM1 was also found to belong to a complex containing several BER proteins, including APE1, Fen1, Polβ and LigI [62, 63].

### NPM1 and cancer

**Fig. 6:**
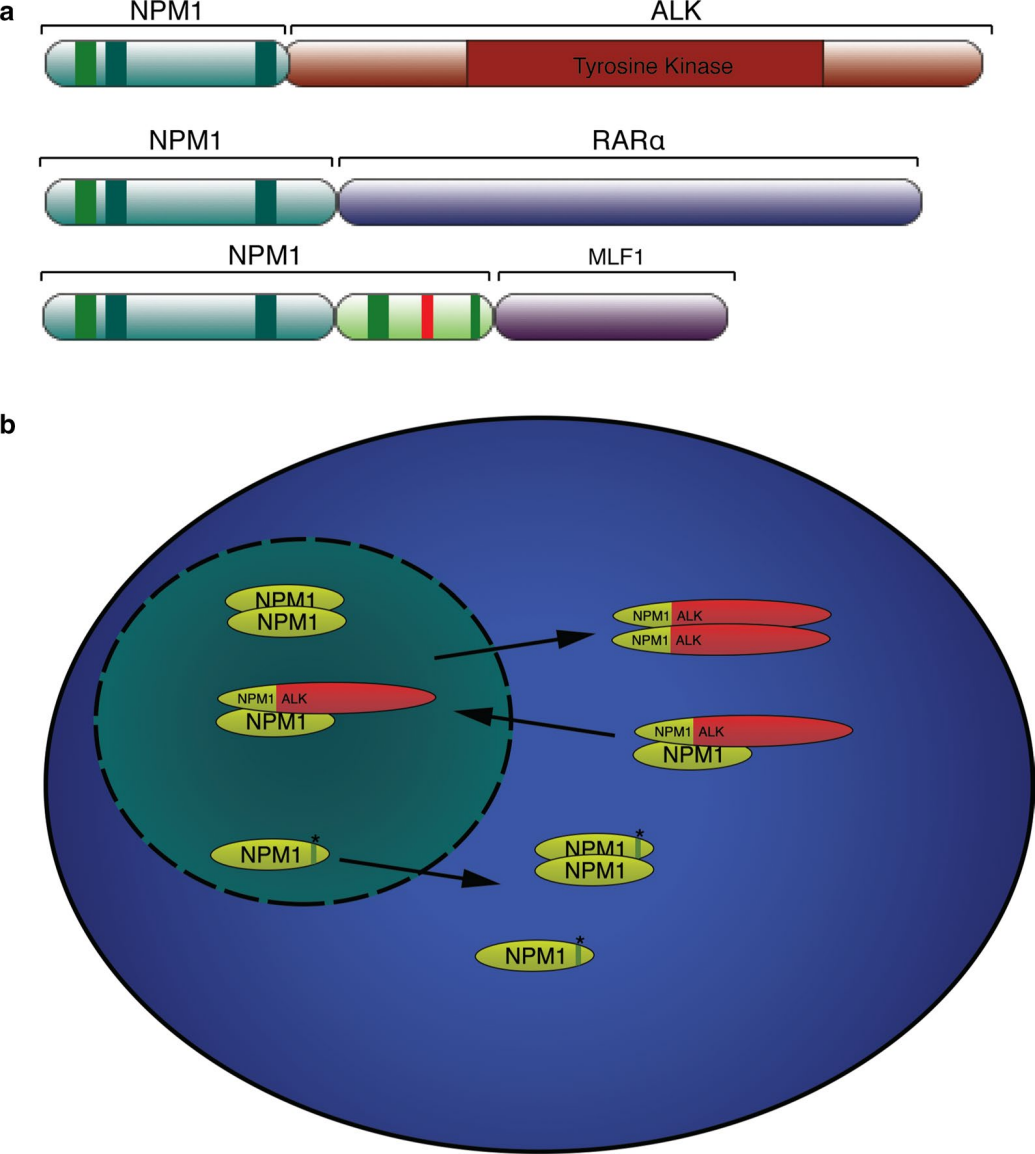
**a** Schematic structure of NPM1 chimeric proteins found in cancers. NPM1 is represented as in Fig. 2b. **b** Representation of the localization of wild-type NPM1, NPM1c+ and NPM1-ALK fusion protein in cancer. NPM1c+ (indicated by an *asterisk*) is cytoplasmic and can form dimers with NPM1 WT, retaining it in the cytoplasm. NPM1-ALK can dimerize and localize to the cytoplasm. Inversely, NPM1-ALK can form heterodimers with NPM1 WT, causing ALK dislocation into the nucleus (*arrow*). These cells shown both aberrant localization of ALK in the nucleus, and NPM1 in the cytoplasm

As discussed above, NPM1 is involved in maintaining genome stability and regulating apoptosis, and, as such, has been described as having both oncogenic and tumor suppressive functions. NPM1 was first associated with cancer where approximately one-third of anaplastic large-cell non-Hodgkin’s lymphomas were found to express a fusion between NPM1 and the catalytic domain of anaplastic lymphoma receptor tyrosine kinase (ALK) [64]. In addition, 35 % of all AML patients (50–60 % in adults with normal karyotype) show NPM1 rearrangements or mutations [65], leading the World Health Organization to introduce mutated NPM1 as an AML entity [66]. All NPM1 mutations reported occur with the C-terminus of the protein altering either the folding of this region or the Nucleolar localization signal itself. Named NPM1 Cytoplasm positive or NPM1c+, these mutations result in cytoplasmic localization of the protein, and act as dominant negative by retaining WT NPM1 in the cytoplasm (Fig. 6b) [67].

Patients with NPM1 mutations proved to have a better outcome with increased complete remission and improved overall survival [68]. However, within this group of patients, the antigen expression pattern of HLADR(+) CD34(+) CD7(+) is associated with poor prognosis [69]. NPM1c+ has been shown to result in microRNAs deregulation [70], and a recent in vitro study established that NPM1c+ could enhance leukemia cells adhesion, migratory and invasive potential through MEK/ERK activation [71]. Notably, NPM1 mutation in a knock-in mouse model resulted in AML initiation [72, 73], and studies on MEFs have shown that NPM1 mutation results in p14^ARF^ destabilization [74] and c-Myc stabilization [75].

In anaplastic large-cell lymphoma (ALCL), NPM1 fusion with anaplastic lymphoma receptor tyrosine kinase (ALK) can be found in 85 % of the ALK+ ALCL, this results in the expression of a chimeric oncogenic protein formed by the C-terminus of NPM1 and the kinase domain of ALK (Fig. 6a) [64, 76]. In these cells both NPM1 and ALK exhibits aberrant localization. Further, dimerization of NPM1-ALK leads to a constitutive activation of the ALK kinase (Fig. 6b) [76].

Chimeric proteins between NPM1 and the retinoic acid receptor-α gene (RARα) or between NPM1 and the myelodysplasia/myeloid leukemia factor 1 (MLF1) have also been reported in rare cases of leukemia (Fig. 6a) [77, 78].

Besides leukemia, involvement of NPM1 has also been reported in several solid cancers. Overall, NPM1 overexpression is linked to high grade tumors and poor prognosis, as observed in brain glioblastoma [79], oral squamous cell carcinoma [80], non-small cells lung cancer (NSCLC) [51], hepatocellular carcinomas (HCC) [81], colon cancer [82–84], ovarian cancer [62, 85], and endometrial carcinoma [86]. A higher level of NPM1 has also been observed in prostate cancer, when compared to normal tissue [87, 88], promoting cells growth and invasiveness [89]. In bladder cancer, high levels of NPM1are not associated with tumor grade, but with cancer progression, recurrence, and poor prognosis [90]. NPM1 overexpression has also been reported in thyroid cancer [91], with thyroid cancer cell lines showing NPM1 mislocalization in the absence of the mutations observed in AML patients [92].

However, low levels of NPM1 have also been observed in some cancers, such as gastric cancers (mRNA and protein) compared to normal tissue [93]. In breast cancer, low NPM1 levels are also associated with poor outcome, independently of the molecular subtype, with granular staining of NPM1 correlating with poor prognosis [94].

Since NPM1 is overexpressed in many types of cancer and because of its role in genome stability, it could be a potential target for new cancer therapy strategies. Different molecules have been trialed to induce cell death by destabilizing NPM1 [95–98] or inhibiting its interaction with other DNA repair proteins [99]. Recent studies have focused on combining NPM1 inhibition with a DNA damaging agent, for instance ionizing radiation [51, 100], or a cytotoxic drug [101]. Other strategies are aiming specifically at NPM1c+ itself [102], or in combination with damage induction by increasing oxidative stress in cells [103–106].

### Frontiers

As summarized here, NPM1 plays multiple roles within human cells while the best documented is in RNA transport and ribosome biogenesis, it is clear that NPM1 also plays a critical role in the regulation of apoptosis and in the maintenance of genomic homeostasis.

Genome instability is one of the underlying causes of cellular transformation and cancer development. Once a cancer does form, genome instability becomes a common feature seen as a universal hallmark of all cancers [107]. This instability provides the cancer the ability to evolve and adapt to the environment in which it is located; further, defects in the apoptosis pathways allow the cancer cell to cope and survive with levels of genetic instability that would normally induce cell death.

Given the critical role NPM1 plays in genome stability and apoptosis it is hardly surprising that NPM1 dysfunction is a frequent feature in cancers. Indeed, as evidence of NPM1 function in DNA repair pathways increases, it explains at least in part the genetic instability associated with cancers such as AML. Further, NPM1 deregulation or mutation will suppress the ability of that cell to respond to apoptotic stimuli, allowing for the tolerance of the genetic instability. Consistent with a role in genetic instability and specifically the repair of double-strand DNA breaks we see that AML patients with a mutated NPM1 have a >2 fold higher odds of achieving complete remission as compared to patients with a wild type NPM1 [108]. NPM1 in cancer is most strikingly highlighted in acute myeloid leukemia where approximately 30 % of patients will have a mutation or fusion event implicating NPM1.

Despite the importance of NPM1 in genome stability it is clear that we do not fully understand how NPM1 functions in the repair of DNA damage. We know that NPM1 moves to sites of double-strand DNA breaks within the genome, but we do not yet understand how NPM1 functions at those break sites. We do not know if NPM1 binds to nucleic acids at those sites or if it is involved in remodeling chromatin. It is clear, however, that understanding how NPM1 participates in DNA repair and genome stability will help to delineate the role of NPM1 in both normal and cancerous cells. This will perhaps provide insights into why NPM1 dysfunction is a marker of drug and radiation sensitivities.

## Abbreviations

AKT: protein kinase B
ALK: anaplastic lymphoma receptor tyrosine kinase
AML: acute myeloid leukemia
APE1: apurinic/apyrimidinic endonuclease 1
ATM: ataxia telangiectasia mutated protein
ATP: adenosine triphosphate
ATR: ataxia telangiectasia and Rad3 related protein
BRCA1: breast cancer 1
CDK: cyclin-dependent kinase
DDB2: damage-specific DNA binding protein 2
DNA: deoxyribonucleic acid
DSB: double-strand breaks
E2F1: E2F transcription factor 1
EDAG: erythroid differentiation-associated gene
HR: homologous recombination
kDa: kilo dalton
MDM2: mouse double minute 2 homolog
MEF: mouse embryonic fibroblast
MLF1: myelodysplasia/myeloid leukemia factor 1
mRNA: messenger RNA
NER: nucleotide excision repair
NHEJ: non-homologous end-joining
NoLS: nucleolar localisation signal
NPM1: nucleophosmin1
PCNA: proliferating cell nuclear antigen
PKB: protein kinase B
RAR: retinoic acid receptor α
RNA: ribonucleic acid
RNF: ring finger protein1
RPA: replication protein A
TLS: translesion DNA synthesis
TNF: tumor necrosis factor
UV: ultra violet
XPC: xeroderma pigmentosum, complementation group C
